# Sliding sampling and successive variational mode decomposition CNN-BiLSTM-attention based fault detection and early warning method for DC microgrid

**DOI:** 10.1038/s41598-026-48188-w

**Published:** 2026-04-15

**Authors:** Yongdong Dai, Maofei Wang, Linghao Zhang, Shenyu Wang, Zhongjun Jiang

**Affiliations:** State Grid Jiangsu Electric Power Co, Ltd, Taizhou Power Supply Branch, Taizhou, 225300 Jiangsu China

**Keywords:** DC microgrid, Fault detection, Sliding sampling, Successive variational mode decomposition, Convolutional neural network, BiLSTM, Attention mechanism, Energy science and technology, Engineering, Mathematics and computing

## Abstract

A fault detection method that integrates sliding sampling, successive variational mode decomposition (SVMD), and CNN-BiLSTM-Attention model is proposed to address the problem of insufficient sensitivity and discrimination ability in fault signal diagnosis of DC microgrids. Firstly, sliding sampling is used to capture transient fault information, avoiding the loss of information in traditional fixed windows; Secondly, by decomposing the fault signal through SVMD, the penalty factor is optimized with the maximum mutual information coefficient (MIC), and the effective modal components (IMF) are selected by combining the correlation coefficient, spectral entropy, and Teager Kaiser energy ratio to achieve noise reduction and signal reconstruction; Finally, a CNN-BiLSTM-Attention classification model is constructed, using CNN to extract local time-frequency features, BiLSTM to capture sequence context relationships, and adaptive weighting of key fault features through attention mechanism to suppress noise interference. The experimental results show that the proposed method has an average classification accuracy of 92.35% in islanding mode and 91.13% in grid connected mode, which is significantly better than the compared methods, especially with an accuracy rate of over 95% in single-phase grounding faults; The accuracy exceeds 91% in different scenarios (radial/mesh topology), verifying its robustness and adaptability.

## Introduction

With the continuous growth of new energy generation scale, the power grid system has increasingly high requirements for the consumption capacity of new energy generation. DC microgrids have become one of the key technologies for enhancing the consumption capacity of new energy due to their efficient energy transmission capability and flexible topology structure^1^. Transmission line faults are the main threat to the reliable power supply of microgrid systems. In order to maintain the stable and reliable operation of microgrid systems and avoid power supply interruptions for users, it is necessary to promptly identify and repair transmission line faults. Therefore, researching high-precision fault diagnosis methods for microgrid systems and reducing relay protection misoperation rates has important engineering value.

DC microgrids have more complex fault signals compared to AC systems^2^: (1) Due to the lack of natural zero crossing of current, the current will rapidly rise and maintain a high level in milliseconds after a fault occurs; (2) Due to the influence of power electronic switch harmonics and renewable energy fluctuations, the signal exhibits strong non stationarity and is overlaid with multi-source noise interference. Therefore, the fault diagnosis of DC microgrids needs to be completed in a short period of time and maintain the accuracy and reliability of diagnosis under multi-source noise interference. In engineering practice, commonly used DC microgrid fault detection methods include differential protection on the basis of current discrimination at both ends, traveling wave protection using fault transient wavefront positioning, and arc sensor protection triggered by optical signals. Although these methods respond quickly and are easy to implement, their detection sensitivity and discrimination ability are still limited in strong noise environments. Existing methods often rely on deep learning or simply combine signal decomposition, but their robustness is insufficient under strong noise conditions^3^. To address this issue, a DC microgrid fault diagnosis method combining optimized SVMD and CNN-BiLSTM-Attention mechanism is proposed, which takes into account the advantages of signal decomposition and deep learning. In the signal decomposition process, SVMD is significantly better than EMD and VMD in avoiding mode mixing^4^. In the classification modeling stage, CNN can extract local time-frequency features, but lacks the ability to characterize sequence dependencies^5^. Bidirectional Long Short Term Memory Network (BiLSTM) is a deep learning model that extends from Long Short Term Memory Network (LSTM), aiming to solve the problem of traditional LSTM only considering past information. By simultaneously utilizing the forward and backward information of sequence data, it significantly improves the model’s ability to capture context relationships^6^. However, in complex noisy environments, relying solely on CNN-BiLSTM may still result in key information being masked. Therefore, on the basis of the CNN-BiLSTM model, attention mechanism is developed to highlight the key signals at the moment of fault through adaptive weighting and suppress noise interference, thereby improving the classification performance under low signal-to-noise ratio. Among the existing hybrid fault diagnosis methods on the basis of signal decomposition and deep learning, the VMD-CNN-LSTM method combines signal decomposition and deep feature extraction. However, the penalty factor of VMD is mostly set on the basis of experience, which can easily lead to mode aliasing or over decomposition problems. Moreover, LSTM can only capture sequence features unidirectionally, and the bidirectional contextual relationship representation of fault transients is insufficient; The wavelet CNN method is limited by the selection of wavelet basis functions and has poor adaptability to strong non-stationary and multi noise superimposed fault signals in DC microgrids, making it difficult to accurately extract the essential characteristics of faults. In response to the common deficiencies mentioned above, this article proposes a sliding sampling optimization SVMD-CNN-BiLSTM Attention integrated fault diagnosis method, which optimizes the entire process from signal acquisition, decomposition and reconstruction, and feature recognition, forming a fault diagnosis framework that combines information capture integrity, signal decomposition accuracy, and targeted feature recognition.

The main work of this study is as follows: (1) Propose an optimized SVMD method that uses the maximum information coefficient (MIC) as the fitness function to determine the optimal penalty factor, and combines the correlation coefficient CC, spectral entropy SpEn, and Teager Kaiser energy ratio TKE to screen fault related IMFs, achieving effective denoising and signal reconstruction. This solves the problem of traditional VMD parameters not being optimized and IMF screening being single. Compared with wavelet decomposition, it has the theoretical advantages of no basis function selection restrictions and more accurate decomposition of non-stationary signals; (2) Build a CNN-BiLSTM Attention classification model, introduce attention mechanism on the basis of CNN-BiLSTM, enhance the model’s perception and weight allocation ability for key fault features, compensate for the shortcomings of traditional CNN LSTM without feature weighting and one-way capture of sequence features, and solve the problem of insufficient capture of transient key fault information in existing fusion models; (3) Build a simulation model of DC microgrid, conduct grounding and short-circuit fault diagnosis experiments, verify the adaptability of the proposed method in different noise environments and operating modes, and compare it with different fitness functions and traditional hybrid methods (VMD-CNN-LSTM, wavelet CNN) to clarify the performance advantages of the proposed method in classification accuracy and noise resistance.

## Fault diagnosis model for DC microgrid

### Fault data sliding sampling

**Fig. 1:**
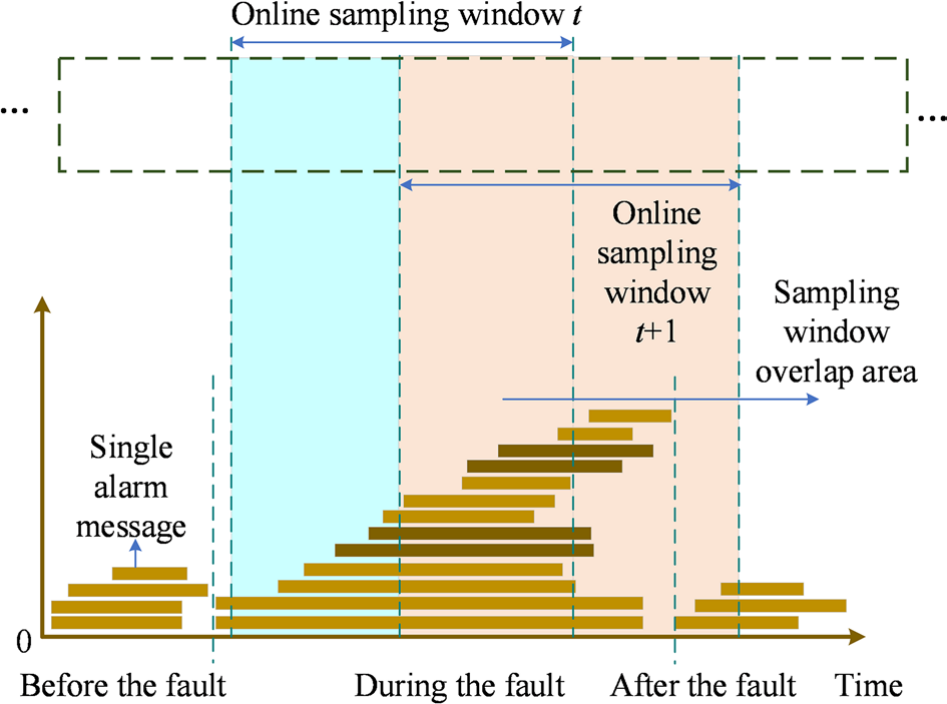
Capturing alarm information using a sliding sampling time window.

The alarm information in the engineering site is transmitted in real-time, and the fault events are randomly distributed within the sampling window^7,8^. Traditional algorithms require the upper bound of the window to capture the beginning of the fault event, and have strict requirements for the sampling time window, that is, the upper bound of the sampling time window corresponds exactly to the start alarm information of the fault event. If this condition is not met, it may not be possible to accurately judge the faulty equipment. This article uses sliding sampling method to collect power grid fault data, as shown in Fig. 1. The horizontal axis in Fig. 1 represents the time when the fault event occurred, and the shaded area indicates the overlapping area of two adjacent sampling time windows^9^.

The process of capturing alarm information in the sliding sampling time window shown in Fig. 1 is as follows^10^: (1) Define the sliding window parameters. (1) The window length should be sufficient to contain critical transient information after a fault occurs, while avoiding excessive length that may increase computational complexity and decrease real-time performance. (2) The sliding step size determines the interval size of window movement, which needs to be balanced and selected according to the actual application scenario. (2) Sliding sampling process. (1) Starting from the starting position of the data stream, initialize the starting and ending points of the first sampling window. (2) Collect current and voltage signal data of DC microgrids within each sampling window for fault feature extraction and classification. (3) After completing the data collection of a window, move the window backwards according to the set sliding step size to form a new sampling window until it covers the entire data stream. (3) Overlapping area design. To avoid information loss caused by window switching, a certain overlap area is set between adjacent sampling windows. In the overlapping area, data will be collected and processed simultaneously by two adjacent windows. (4) Alarm information capture. Real time monitoring of signal characteristics within each sampling window during sliding sampling process. Once abnormal signal characteristics such as current and voltage are detected, the alarm mechanism is immediately triggered.

### Sequential variational mode decomposition

The fault current of DC microgrids has short-term abrupt changes and non-stationary characteristics. Traditional EMD and VMD are prone to mode mixing and over/under decomposition, resulting in weakened transient details and residual noise. To this end, SVMD is used to decompose the signal under limited bandwidth constraints, and the spectral energy concentration of the modes is improved by updating the center frequency through proxy transmission, thereby obtaining a bandwidth limited and center frequency adaptive modal representation^11^. SVMD decomposes the original signal into several IMFs by solving a constrained variational optimization problem. The core of SVMD is to achieve spectral energy concentration and use penalty factors to suppress aliasing, so that the energy of each mode is mainly distributed in the corresponding sub-band. During the solving process, Lagrange multiplier method is used to alternately update the mode and center frequency, which can reduce aliasing, improve decomposition stability and reconstruction quality. The optimization objective can be expressed as^12^:

1 $${P_{{\mathrm{SVMD}}}}=\hbox{min} \sum\limits_{{k=1}}^{K} {{{\left\| {{\partial _t}\left( {{u_k}} \right)} \right\|}^2}} +\alpha \sum\limits_{{k=1}}^{K} {{{\left\| {{u_k} - f} \right\|}^2}}$$

Where, *α* is the penalty factor; ${u_k}$ is the decomposed modal component; *f* is the original signal.

The fitness function is used to measure the quality of decomposed features, and its design directly affects the optimization performance and final decomposition quality. The commonly used fitness functions include^13,14^: (1) envelope kurtosis factor EK (envelope kurtosis factor), which measures the pulse characteristics of the signal envelope; (2) The envelope spectrum peak factor (ESP) evaluates the sharpness of the signal envelope spectrum; (3) Pearson correlation coefficient PCC (Pearson correlation coefficient) reflects the linear correlation between signals; (4) The Maximum Information Coefficient (MIC) measures the nonlinear dependence between signals. Due to MIC’s superior ability to capture nonlinear dependencies, robustness to noise and outliers, and stronger compatibility with SVMD decomposition targets compared to other fitness functions, this study selects the maximum mutual information coefficient as the fitness function^15^.

### IMF feature screening indicators

After SVMD optimization decomposition, some IMF may carry a lot of noise or residual information, and multiple feature evaluation indicators need to be combined to screen IMF.

1. Correlation coefficient. Measure the similarity between IMF and the original signal.
  2 $$CC={{\sum\limits_{{k=1}}^{K} {\left( {{X_k} - \bar {X}} \right)} \left( {{Y_k} - \bar {Y}} \right)} \mathord{\left/ {\vphantom {{\sum\limits_{{k=1}}^{K} {\left( {{X_k} - \bar {X}} \right)} \left( {{Y_k} - \bar {Y}} \right)} {\left( {\sqrt {\sum\limits_{{k=1}}^{K} {{{\left( {{X_k} - \bar {X}} \right)}^2}} } \cdot \sqrt {\sum\limits_{{k=1}}^{K} {{{\left( {{Y_k} - \bar {Y}} \right)}^2}} } } \right)}}} \right. \kern-0pt} {\left( {\sqrt {\sum\limits_{{k=1}}^{K} {{{\left( {{X_k} - \bar {X}} \right)}^2}} } \cdot \sqrt {\sum\limits_{{k=1}}^{K} {{{\left( {{Y_k} - \bar {Y}} \right)}^2}} } } \right)}}$$
  Where, $\bar {X}$ and $\bar {Y}$ are the mean values of signals *X* and *Y*, respectively.
2. Spectral entropy. Reflect the complexity of the signal spectrum.
  3 $$SpEn= - \sum\limits_{{i=1}}^{N} {{P_i}} \lg {P_i}$$
  Where, ${P_i}$ is the normalized value of signal power spectral density.
3. Teager-Kaiser energy ratio. Measure the transient energy of the signal and identify the characteristics of sudden changes in the signal.
  4 $$TKE=\frac{1}{{N - 2}}\sum\limits_{{n=2}}^{{N - 1}} {\left[ {x{{(n)}^2} - x(n - 1)x(n+1)} \right]}$$
  Where, *N* is the number of sample points; $x(n)$ is the signal sequence.

*CC*, *SpEn*, and *TKE* measure the effectiveness of IMF from three complementary dimensions: morphological similarity, spectral concentration, and transient energy intensity. The effective fault components are usually manifested as high *CC*, low *SpEn*, and high *TKE*; Stable or broadband noise corresponds to low *CC*, high *SpEn*, and low *TKE*; Although peak or pulse interference may increase *TKE*, it is often accompanied by low *CC* or high *SpEn*. Therefore, a joint threshold criterion is adopted: the IMF is retained when $TKE>{\tau _{{\mathrm{TKE}}}}$, $SpEn<{\tau _{{\mathrm{SpEn}}}}$, and $CC>{\tau _{{\mathrm{CC}}}}$.

### Fault diagnosis algorithm framework

**Fig. 2:**
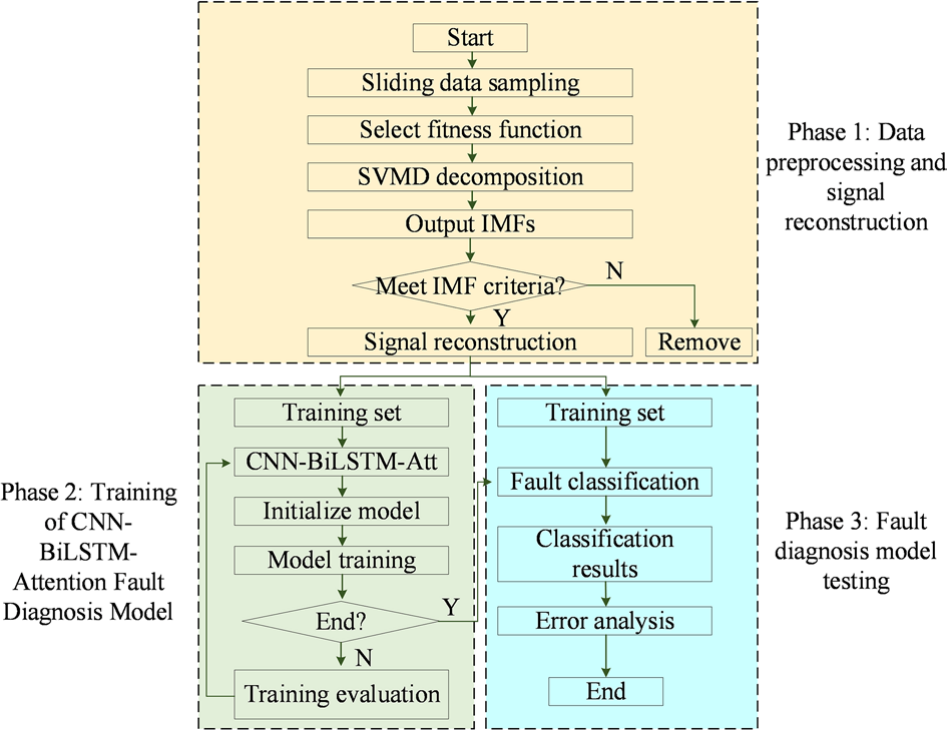
Fault diagnosis algorithm framework.

The fault diagnosis of DC microgrid adopts SVMD optimization to decompose the current signal affected by noise interference, and selects IMF on the basis of the joint criteria of *CC*, *SpEn*, and *TKE*. Then, CNN-BiLSTM Attention completes the fault classification. The overall fault diagnosis process is demonstrated in Fig. 2.

## Fault signal diagnosis on the basis of CNN-BiLSTM ATT

### Model framework

**Fig. 3:**
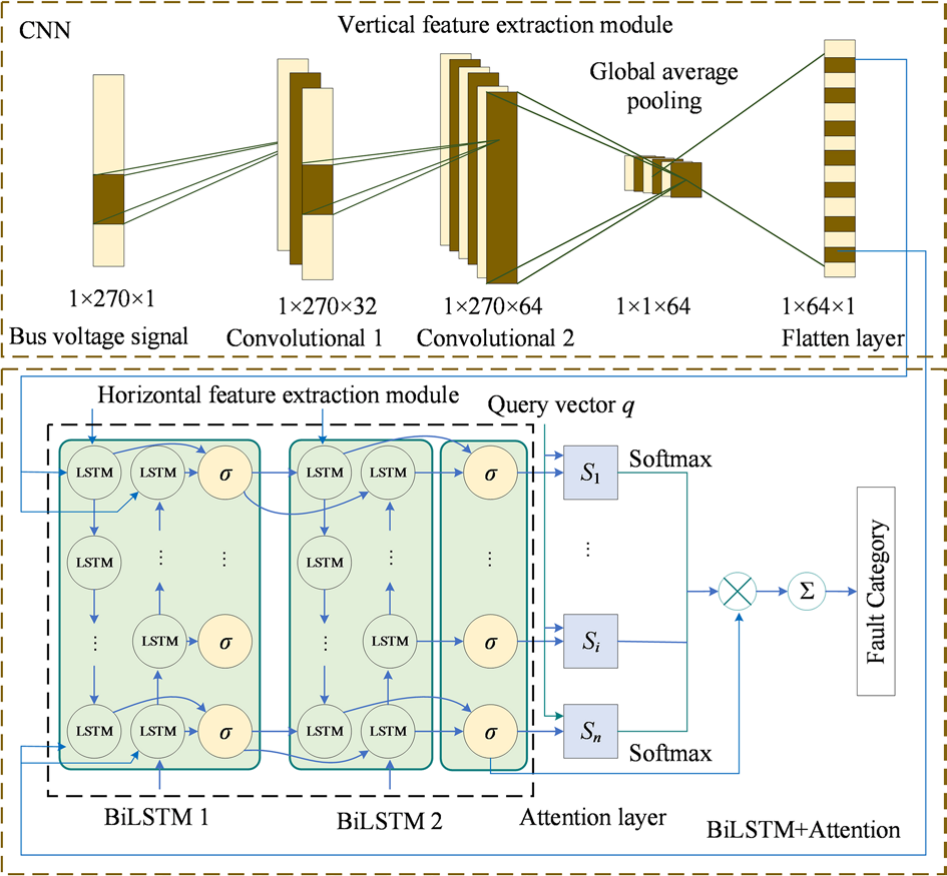
Structure of CNN-BiLSTM.

The structure of the fault diagnosis model on the basis of the CNN-BiLSTM-Attention fusion model is demonstrated in Fig. 3.

For the input of bus voltage signals, the vertical feature processing module with two convolutional layers as the core is first used, and then the horizontal feature processing module with BiLSTM network as the core is used^16^. Finally, by adding attention mechanisms, we can more effectively focus on the changing patterns of data features near the time of fault occurrence^17,18^.

### Vertical feature capture component

The core of the vertical feature capture component is CNN, which can extract detailed features of data. The formula for convolutional procedure using DC microgrid bus voltage data as input is:

5 $$z_{j}^{l}=\sum\limits_{{i=1}}^{m} {z_{j}^{{l - 1}}} \times k_{{ij}}^{l}+b_{j}^{l},j=1, \cdots ,n$$

Where, $z_{j}^{l}$ is the *j*-th feature surface feature vector; $k_{{ij}}^{l}$ is the parameter connecting the *j*-th learnable kernel of the *i*-th input feature surface; $z_{j}^{{l - 1}}$ is the voltage signal of the *i*-th input feature surface; $b_{j}^{l}$ is the bias of the *j*-th learnable kernel.

In model training, data distribution may shift, and normalization can be used to avoid gradient vanishing. The standardization function is as follows:

6 $${z_{{\mathrm{LN}}}}=\frac{{z - E(z)}}{{\sqrt {{V_{{\mathrm{ar}}}}(z)+\epsilon } }} \times \alpha +\beta$$

Where, $E(z)$ is the mean value; *z* is the result produced by voltage signal convolutional procedure; ${V_{{\mathrm{ar}}}}(z)$ is the variance value; *ε* is generally taken as 10^− 5^ to prevent a zero-valued denominator; *α*, *β* is a trainable parameter.

The purpose of the activation operator is to excavate nonlinear features inherent in voltage signals and strengthen the approximation capability of the network. The ReLu activation operator used is calculated as follows:

7 $$x={R_{{\mathrm{eLU}}}}\left( {{z_{{\mathrm{LN}}}}} \right)=\left\{ {\begin{array}{*{20}{l}} {{z_{{\mathrm{LN}}}},}&{{z_{{\mathrm{LN}}}} \geqslant 0} \\ {0,}&{{z_{{\mathrm{LN}}}}<0} \end{array}} \right.$$

### Horizontal feature capture component

The core of the horizontal feature capture component is the BiSTM network, which is constituted by a forward sequential LSTM and a backward sequential LSTM^19^. The LSTM unit structure calculation model is as follows:

8 $$\left\{ {\begin{array}{*{20}{l}} {{f_t}=\sigma \left( {{{\boldsymbol{W}}_f} \cdot \left[ {{h_{t - 1}},{x_t}} \right]+{b_f}} \right)} \\ {{i_t}=\sigma \left( {{{\boldsymbol{W}}_i} \cdot \left[ {{h_{t - 1}},{x_t}} \right]+{b_i}} \right)} \\ {{{\tilde {C}}_t}=\tanh \left( {{{\boldsymbol{W}}_{\tilde {C}}} \cdot \left[ {{h_{t - 1}},{x_t}} \right]+{b_{\tilde {C}}}} \right)} \\ {{C_t}={f_t} \otimes {C_{t - 1}} \oplus {i_t} \otimes {{\tilde {C}}_t}} \\ {{o_t}=\sigma \left( {{{\boldsymbol{W}}_o} \cdot \left[ {{h_{t - 1}},{x_t}} \right]+{b_o}} \right)} \\ {{h_t}={o_t} \otimes \tanh {C_t}} \end{array}} \right.$$

Where, $\otimes$ is element wise multiplication; $\oplus$ is element wise addition; ${C_t}$ is the memory unit; ${\tilde {C}_t}$ is a temporary memory unit; ${x_t}$ is the input; ${h_t}$ is the hidden layer output; The forget gate determines which information is added to the current memory unit ${C_t}$, while the output gate controls the output result of the memory unit ${C_t}$. ${{\boldsymbol{W}}_f}$, ${{\boldsymbol{W}}_i}$, ${{\boldsymbol{W}}_{\tilde {C}}}$, ${{\boldsymbol{W}}_o}$ are the weight matrices corresponding to each module; ${b_f}$, ${b_i}$, ${b_{\tilde {C}}}$, and ${b_o}$ are bias terms; $\sigma$ is the sigmoid activation operator; tanh is the hyperbolic tangent activation operator.

**Fig. 4:**
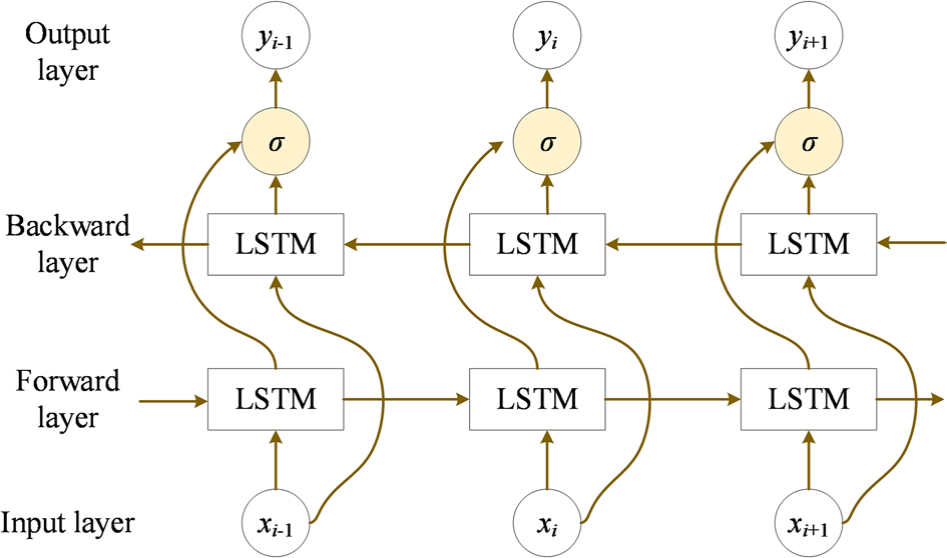
BiLSTM Structure.

The BiLSTM network combines forward LSTM and backward LSTM, and more effectively explore the intrinsic connections between current time data and past and future time data. The BiLSTM structure is demonstrated in Fig. 4.

The BiLSTM network calculation model is^20^:

9 $$\left\{ {\begin{array}{*{20}{l}} {{{\vec {h}}_t}=\operatorname{LSTM} \left( {{x_t},{{\vec {h}}_{t - 1}}} \right)} \\ {{{\overset{\lower0.5em\hbox{$\smash{\scriptscriptstyle\leftarrow}$}}{h} }_t}=\operatorname{LSTM} \left( {{x_t},{{\overset{\lower0.5em\hbox{$\smash{\scriptscriptstyle\leftarrow}$}}{h} }_{t - 1}}} \right)} \\ {{y_t}=\sigma \left( {{{\boldsymbol{W}}_y} \cdot \left[ {{{\vec {h}}_t},\bar {h}} \right]+{b_y}} \right)} \end{array}} \right.$$

Where, LSTM() is a computational model for LSTM networks; $${{\boldsymbol{W}}_y}$$ is the weight matrix; $${\vec {h}_t}$$ represents the forward hidden layer state at time *t*; $${\overset{\lower0.5em\hbox{$\smash{\scriptscriptstyle\leftarrow}$}}{h} _t}$$ is in the backward hidden layer state; $${b_y}$$ is the bias term.

### Attention mechanism

To improve the estimation performance of BiLSTM model, attention module is developed to optimize BiLSTM, aiming to capture the highly relevant information through attention weights rather than global information. When forecasting the output sequence, the attention module will pay more attention to the key data in the input sequence of the BiLSTM model, allowing the network to focus on specific information.

The input sequence is ${y_1},{y_2}, \cdots ,{y_t}$, and the corresponding hidden state is ${{\boldsymbol{h}}_1},{{\boldsymbol{h}}_2}, \cdots ,{{\boldsymbol{h}}_t}$. The calculation formula is:

10 $${{\boldsymbol{S}}_{ti}}=\tanh \left( {{\boldsymbol{W}}{h_t}+{\boldsymbol{U}}{h_i}+{\boldsymbol{b}}} \right)$$

11 $${{\boldsymbol{a}}_{ti}}={{\exp \left( {{{\boldsymbol{S}}_{ti}}} \right)} \mathord{\left/ {\vphantom {{\exp \left( {{{\boldsymbol{S}}_{ti}}} \right)} {\sum\limits_{{k=1}}^{t} {\exp } \left( {{{\boldsymbol{S}}_{tk}}} \right)}}} \right. \kern-0pt} {\sum\limits_{{k=1}}^{t} {\exp } \left( {{{\boldsymbol{S}}_{tk}}} \right)}}$$

12 $${\boldsymbol{F}}=\sum\limits_{{i=1}}^{t} {{{\boldsymbol{a}}_{ti}}} \times {{\boldsymbol{a}}_i}$$

13 $${{\boldsymbol{h}}^{\prime}_t}=f\left( {{\boldsymbol{F}},{{\boldsymbol{h}}_t},{y_t}} \right)$$

Where, ${{\boldsymbol{S}}_{ti}}$ represents the attention score of each hidden layer for the final output of the model, which is transformed into attention weights using the Softmax function. The attention weight of the *i*-th element is ${{\boldsymbol{a}}_{ti}}$; ${\boldsymbol{F}}$ is the weighted sum of attention weights and corresponding input hidden state values ${{\boldsymbol{h}}_i}$; ${{\boldsymbol{h}}^{\prime}_t}$ is the final feature vector. ${\boldsymbol{W}}$, ${\boldsymbol{U}}$, and ${\boldsymbol{b}}$ are model learning parameters that are dynamically adjusted during the model training process.

## Experimental analysis

### Construction of DC microgrid system

**Fig. 5:**
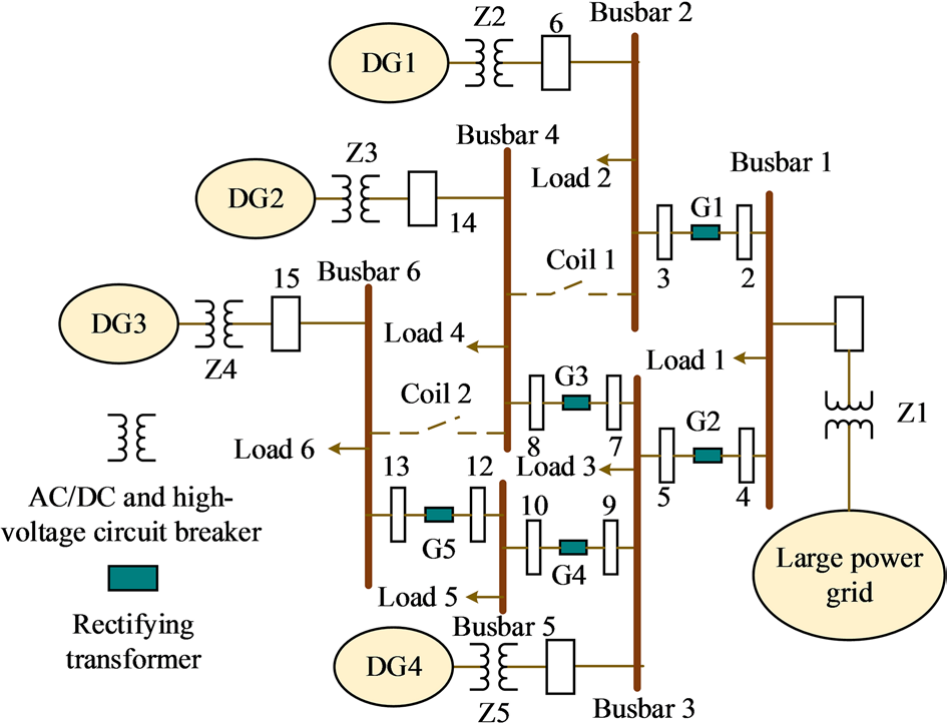
Topology structure of DC microgrid system.

A simulation model on the basis of actual operating parameters was established using the Xinjiang wind solar hydrogen production project as a prototype, as shown in Fig. 5, and the parameters are listed in Table 1. Equipment and line parameter selection: The bus voltage level should be consistent with the commonly used voltage level of the new energy grid connected device to ensure compatibility with the actual DC distribution system; The rated capacity of photovoltaic and wind power refers to the single unit scale setting of the project to ensure that it matches the load and energy storage capacity power; The energy storage capacity is on the basis of the configuration of the lithium battery energy storage unit in the project, ensuring that it has the functions of regulation and peak valley filling; The setting of DC and AC loads meets the load requirements under typical operating conditions; The line parameters and a length of 2 km are selected from commonly used cable configurations in the region to reflect the actual transient transmission characteristics.

The experiment was conducted in the Win10 Professional operating system environment, with a computing device configuration of 32GB of memory, and the deep learning part was implemented on the basis of MATLAB 2022b’s Deep Learning Toolbox. Build a DC microgrid model as shown in Fig. 5 on the Simulink platform and generate a dataset through batch simulation using sliding window sampling. The duration of a single simulation is 2 s, with a sampling frequency of 8 kHz. All faults are set to occur at 1.50 s, and the bus current sequence is captured as a single sample (960 points) within the time window of [1.48 s, 1.60 s]. The data includes three types of operating conditions: normal, grounded, and short-circuit. Each type of working condition generates 300 independent scenarios through stratified randomization, and adds 4 independent noise seeds to each scenario to obtain 1200 sets of samples, totaling 3600 sets for the 3 types of working conditions. To simulate the actual operating environment of DC microgrids, three typical types of noise interference are added to the simulation signal: random noise (mainly from measurement and sensors), high-frequency switch harmonics (caused by the switching action of power electronic converters), and transient pulse interference (mostly seen in high-power load switching). Three types of noise are superimposed, and the signal-to-noise ratio (SNR) is set to 10dB to quantify the overall noise intensity.

**Table 1:** Parameters of DC microgrid system.

Name	Parameter
Bus voltage	± 500 V
photovoltaic system	40 kW, MPPT control, solar irradiance of 1000 W/m, temperature of 25 ℃, DC-DC boost converter
Wind power system	24 kW, PMSG, Wind speed 12 m
Energy storage system	384 V, 50Ah, 60kWDC-DC bidirectional buck boost converter
Public power grid	50 Hz, 380 V, 200 kW, Grid connected controller
DC load	220 V, 20 kW, DC-DC buck converter
Communication load	50 Hz, 380 V, 4 × 8 kW, DC-AC converter
Line parameters	Resistance 0.7180Ω/km, inductance 0.357mH/km, capacitance 0.523 µF/km, length 2 km

Using data augmentation techniques to expand fault samples: for current and voltage fault signals, three data augmentation methods were introduced: amplitude perturbation (± 5%), time axis shift (± 2 sampling points), and weak Gaussian white noise superposition (SNR=30dB). The original samples of each type of fault were expanded fourfold, resulting in a final experimental dataset of 14,400 sets, including 4800 sets for normal operating conditions, 4800 sets for ground faults, and 4800 sets for short-circuit faults. During the data augmentation process, it is strictly ensured that the fault features of the samples remain unchanged, and only non-core features are perturbed. This study used a double validation strategy to conduct experiments: repeated experiments with multiple random seeds. Set 10 different random seeds (1, 23, 45, 67, 89, 101, 123, 145, 167, 189) and randomly partition the expanded 14,400 dataset 10 times on the basis of a 70/30 ratio. Calculate the average classification accuracy (ACA) and standard deviation (SD) of the 10 experimental results to verify the stability of the model under different data partitions.

### Evaluation indicators

To evaluate the effectiveness of the proposed method, a data sample list was used for testing, where 1800 samples were selected from the current and voltage signals of a single dataset, and 30% of the data were randomly selected for performance validation. Meanwhile, select Average Classification Accuracy (ACA), recall, and precision as evaluation metrics. The ACA calculation is as follows^21^:

14 $${\varTheta _{{\mathrm{ACA}}}}=\frac{{TN+TP}}{{TN+TP+FN+FP}}$$

Where, *TP* is true positive, which means that a label is correctly classified by the classifier and belongs to the original label category; *TV* is true negative, which means the label is correctly classified by the classifier and does not belong to the original label category; *FP* is false positive, meaning the classifier classifies the label as the original label type, but it does not belong to the original type; *FV* is a false negative, meaning that a label is classified by the classifier as not of the original label type, but it belongs to the original type.

However, the average classification accuracy cannot reflect the detailed results of the proposed method’s performance. In order to study the performance of the classification method in a single fault type, the F1 value is used to further evaluate the classification performance. The F1 value is a function of precision and recall. When its value is 1, it is considered optimal, while when it is 0, its performance is worst. The calculation of precision ${\varTheta _{{\mathrm{precision}}}}$ and ${\varTheta _{{\mathrm{recall}}}}$ recall rate is as follows:

15 $${\varTheta _{{\mathrm{precision}}}}=\frac{{TP}}{{TP+FP}},{\varTheta _{{\mathrm{recall}}}}=\frac{{TP}}{{TP+FN}}$$

### Analysis of the influence of sampling frequency and signal type

**Fig. 6:**
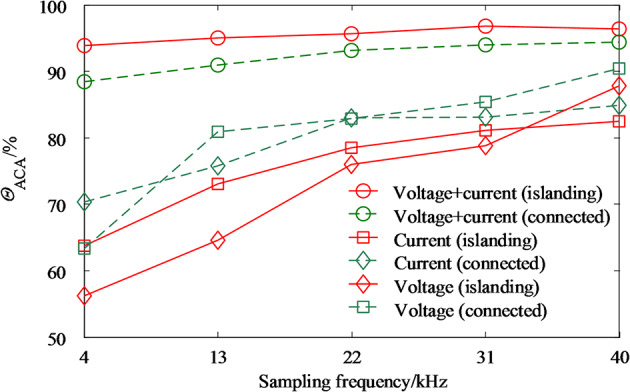
Fault classification accuracy under different sampling frequencies and operating modes.

In the proposed method, current and voltage waveforms are collected at a sampling frequency of 40 kHz. In fact, due to the limitations of data collection in power equipment, the sampling frequency (SF) may be much lower than 40 kHz, and the current and voltage waveforms may not be obtained simultaneously. Therefore, the fault classification performance of the proposed method varies with the input signal type and sampling rate. The ACA results under different MG operating modes are shown in Fig. 6, where the MG operating modes are divided into two main modes: islanding and grid connected. The red and black lines represent islanding and grid connected modes, respectively.

From Fig. 6, it can be seen that as the sampling frequency increases, the classification accuracy continues to improve. As the sampling frequency increases, the obtained fault information becomes more detailed, resulting in higher classification accuracy. At smaller sampling frequencies, current waveforms have better classification performance than voltage waveforms, and for different fault categories, voltage waveforms carry less low-frequency fault information than current waveforms. At high sampling frequencies, voltage signals have a greater impact on classification performance, and their voltage waveforms contain backup fault transients, making them suitable for analyzing fault types at higher sampling rates. Therefore, using specific fault information of current and voltage simultaneously can achieve higher fault classification performance, with a classification accuracy of over 91.54%. Therefore, when the sampling frequency is as high as possible and voltage and current signals are used simultaneously, the classification performance of the proposed method is optimal.

### Fault diagnosis and classification performance analysis

**Fig. 7:**
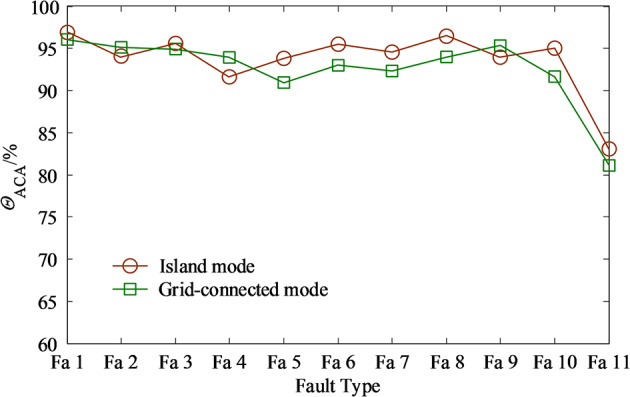
The fault classification accuracy of the proposed method.

The proposed method uses ${\varTheta _{{\mathrm{ACA}}}}$ to evaluate its classification accuracy in 11 types of faults, including DC bus short circuit fault (Fa 1), DC bus polarity grounding fault (Fa 2), high resistance grounding fault (Fa 3), line disconnection/open circuit fault (Fa 4), photovoltaic array fault (Fa 5), energy storage battery fault (Fa 6), DC/DC converter fault (Fa 7), bidirectional AC/DC converter fault (Fa 8), load side fault (Fa 9), passive component faults such as capacitor/inductor (Fa 10), and communication and sensor fault (Fa 11). This demonstrates its fault detection and classification performance, which includes two MG operation modes: grid connected and islanded. The results are shown in Fig. 7.

From Fig. 7, it can be seen that the overall accuracy of the proposed method is higher in island mode, with an initial fault (Fa 1) accuracy of 97% and most fault types maintained between 92% and 97%; The initial accuracy of the grid connected mode is 96%, with a slightly larger overall fluctuation range of 91% to 96%. Comparing the two modes, the islanding mode has significant advantages in faults such as Fa 6 and Fa 8, reaching 96% and 97% respectively, while the grid connected mode performs better in Fa 9 fault (96%), and the two perform similarly in Fa 1 and Fa 3 faults. In the most complex communication and sensor faults (Fa 11), both modes showed a significant decrease in accuracy, with islanding mode dropping to 83% and grid connected mode dropping to 81%, but still maintaining above 80%, indicating that the method still has reliable identification capabilities for extreme faults.

Communication and sensor faults (Fa11) have become the type of fault with the lowest classification accuracy. The core reason is that: (1) this fault is not a physical fault on the power side of the DC microgrid, but an abnormality in the signal acquisition and transmission process. Its characteristics in current and voltage signals show no obvious transient characteristics, and the difference from normal operating signals is much lower than that of power side faults such as grounding and short circuits; (2) Sensor noise and communication interference can superimpose with the random noise and high-frequency switch harmonics of the system itself, further masking weak fault features. Even after SVMD optimization decomposition and multi-index IMF screening, it is still difficult to completely separate pure fault features, ultimately resulting in a decrease in the recognition accuracy of the classification model for the fault. DC side faults such as Fa1-Fa3 have strong transient characteristics, and when a fault occurs, the current and voltage will sharply rise/fall in milliseconds, with high feature recognition. Moreover, the distribution of such fault features in the frequency domain is more concentrated, and SVMD can accurately decompose the corresponding effective IMF, thus maintaining a high recognition accuracy of over 94%. In addition, the overall accuracy of fault classification in grid connected mode is slightly lower than that in islanding mode. The reason is that there is power interaction between the DC microgrid and the utility grid during grid connected operation, and the background noise on the grid side introduces additional signal interference. At the same time, the adjustment action of the grid connected controller will cause a certain degree of attenuation of the transient characteristics of the fault. In islanding mode, the system operates independently, and the propagation of fault characteristics is more direct, with less external interference. Therefore, the model is more accurate in identifying faults in islanding mode.

**Fig. 8:**
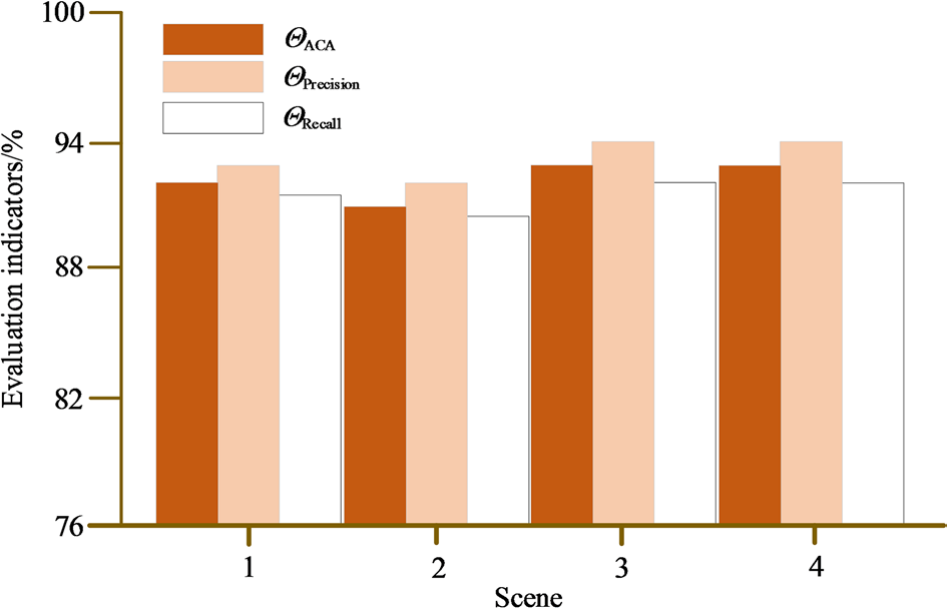
Accurate fault classification in different scenarios.

Overall, the proposed method has an average accuracy rate of over 92% for 11 types of faults in both operating modes, with an average accuracy rate of about 94.2% in islanding mode and about 93.1% in grid connected mode, verifying its robustness and generalization in different operating scenarios. In addition, on the basis of different MG operating modes, combined with different system topologies (radial and mesh), four scenarios are formed. Scenario 1: Mesh topology + islanding mode; Scenario 2: Radial topology + islanding mode; Scenario 3: Mesh topology + grid connected mode; Scenario 4: Radial topology + grid connected mode, the accuracy of fault classification in different scenarios is demonstrated in Fig. 8.

From Fig. 8, it can be seen that the proposed method maintains high classification accuracy in all four scenarios, with ${\varTheta _{{\mathrm{ACA}}}}$ values exceeding 91%. Due to the combination of radial topology and islanding operation mode in scenario 2, the system stability is poor due to the influence of distributed energy, resulting in poor overall performance. Scenario 4 is a combination of mesh architecture and grid connected mode, and MG is relatively stable, so the accuracy of fault diagnosis and classification is ideal, close to 94%.

### Horizontal comparison of algorithms

In order to demonstrate the performance of the proposed method (island mode), it was compared and analyzed with references^22,23^. At the same time, to verify the performance advantages of the proposed framework compared to existing hybrid methods, two hybrid methods, VMD-CNN-LSTM and wavelet CNN, were selected for information comparison. The comparison results of the classification accuracy of the three methods for 11 types of faults are shown in Table 2.

**Table 2:** Comparison of classification accuracy (10 random seed experiments).

Method	${\varTheta _{{\mathrm{ACA}}}}$/%				
	Reference^22^	Reference^23^	VMD-CNN-LSTM	Wavelet-CNN	Proposed method
Fa 1	85.84 ± 1.25	91.45 ± 0.98	94.35 ± 0.65	93.38 ± 0.72	97.32 ± 0.25
Fa 2	83.48 ± 1.32	90.24 ± 1.05	93.28 ± 0.72	92.19 ± 0.78	94.65 ± 0.32
Fa 3	85.83 ± 1.18	92.59 ± 0.92	95.01 ± 0.62	94.57 ± 0.68	95.17 ± 0.28
Fa 4	80.57 ± 1.45	87.10 ± 1.15	91.28 ± 0.85	90.45 ± 0.92	92.08 ± 0.35
Fa 5	82.06 ± 1.38	89.39 ± 1.02	92.60 ± 0.78	91.03 ± 0.85	94.53 ± 0.30
Fa 6	81.00 ± 1.52	88.15 ± 1.18	92.17 ± 0.82	90.89 ± 0.90	96.82 ± 0.22
Fa 7	77.02 ± 1.65	83.79 ± 1.32	89.32 ± 0.95	88.25 ± 1.05	95.69 ± 0.28
Fa 8	77.14 ± 1.61	83.42 ± 1.35	86.49 ± 1.02	84.36 ± 1.12	97.48 ± 0.20
Fa 9	77.30 ± 1.58	84.67 ± 1.28	87.21 ± 0.98	85.09 ± 1.08	94.91 ± 0.32
Fa 10	67.69 ± 2.15	65.03 ± 2.32	81.94 ± 1.25	79.85 ± 1.32	95.32 ± 0.25
Fa 11	69.85 ± 2.08	67.76 ± 2.25	78.93 ± 1.38	75.43 ± 1.55	83.16 ± 0.45

From Table 2, it can be seen that: (1) In the DC side faults (Fa1-Fa3), the accuracy of the proposed method is 97.32%, 94.65%, and 95.17%, respectively, which are 11.48%, 11.17%, and 9.34% higher than literature^22^, 5.87%, 4.41%, and 2.58% higher than literature^23^, 3.94%, 2.46%, and 0.60% higher than wavelet CNN, and 2.97%, 1.37%, and 0.16% higher than VMD-CNN-LSTM, respectively, accurately adapting to the feature recognition requirements of DC side faults. (2) In power electronics and energy storage related faults (Fa4-Fa8), the advantages of the proposed method are more prominent: the accuracy of Fa8 reaches 97.48%, which is 20.34% and 14.06% higher than literature^22,23^ and 10.99% and 13.12% higher than VMD-CNN-LSTM and wavelet CNN, respectively; The accuracy of Fa6 is 96.82%, which is 15.82%, 8.67%, 4.65%, and 5.93% higher than the four comparison methods, respectively; The accuracy of Fa7 is 95.69%, which is the highest improvement of 18.67% compared to the comparative method, fully verifying the feature extraction ability of the proposed method for complex power electronic equipment faults. (3) In load side and passive component faults (Fa9-Fa10), the proposed method solves the problem of insufficient recognition accuracy of traditional methods: the accuracy of Fa10 is 95.32%, which is 27.63% and 30.29% higher than literature^22,23^ and 13.38% and 15.47% higher than the two hybrid methods, respectively; The accuracy of Fa9 is 94.91%, with an improvement range of 7.70% -17.61% compared to the comparative method. Even in the most challenging Fa 11, the proposed method still achieved an accuracy of 83.16%, which is 13.31%, 15.40%, 4.23%, and 7.73% higher than the four compared methods, demonstrating strong robustness. Overall, the proposed method has the best comprehensive performance, highlighting its superiority in identifying multiple faults in islanded microgrids. In addition, the proposed method has the smallest standard deviation (0.20–0.45), demonstrating high stability under 10 random seed experiments; The traditional literature methods^22,]^^23^ have the largest standard deviation (1.18–2.32), while VMD-CNN-LSTM and Wavelet CNN are in the middle, matching the feature extraction ability of the methods themselves.

### Ablation experiment

To verify the independent contributions of core modules such as sliding sampling and SVMD optimization in the proposed method to fault diagnosis performance, ablation experiments were designed. Using the complete model (denoted as M0) as a benchmark, six comparative models were constructed by removing the target module or replacing it with traditional methods. In the islanding operation mode, the effectiveness of each module was quantitatively analyzed using the average classification accuracy (ACA), average F1 value, and average sample inference time of 11 types of faults as evaluation indicators.

If the complete model is marked as M0, the construction method of each comparative model in the ablation experiment is as follows: (1) M1 model. Replace sliding sampling with traditional fixed window sampling, which includes fixed window sampling + optimized SVMD + IMF multi-index screening + CNN-BiLSTM Attention; (2) M2 model. Optimize SVMD and replace it with unoptimized (empirical parameter) VMD, which includes sliding sampling + empirical parameter VMD + IMF multi-index screening + CNN-BiLSTM Attention; (3) M3 model. Replace IMF multi-index screening with single correlation coefficient (CC) screening, which includes sliding sampling + optimized SVMD + single CC screening IMF + CNN-BiLSTM Attention; (4) M4 model. Remove the CNN module and directly input the reconstructed signal into BiLSTM Attention, which includes sliding sampling, optimized SVMD, IMF multi criteria screening, and BiLSTM Attention; (5) M5 model. Remove the BiLSTM module and directly input the CNN output into Attention, which includes sliding sampling + optimized SVMD + IMF multi criteria filtering + CNN Attention; (6) M6 model. Remove the Attention mechanism and retain the CNN-BiLSTM structure, which includes sliding sampling + optimized SVMD + IMF multi criteria screening + CNN-BiLSTM.

On the basis of the analysis of indicator changes, fault diagnosis processes, and the contribution of each module, the comparative results of ablation experiments for each model are shown in Table 3.

According to the ablation experiment data in Table 3, the complete model M0 can achieve a highest ACA of 94.50% and an average F1 value of 0.938. Although the single sample inference time of 18.65ms is slightly higher than other models, it still meets the requirements of millisecond level real-time diagnosis. After removing or replacing each module, the performance of the model decreased to varying degrees, with BiLSTM and CNN modules having the most significant impact. The ACA of M5 and M4 decreased by 7.61% and 7.14%, respectively, and the average F1 value decreased by 8.42% and 7.89%, respectively. This proves that both of them, as the core of feature extraction, are indispensable for extracting local time-frequency features and bidirectional sequence features of faults. The performance degradation of SVMD optimization and IMF multi-index screening module is second, while the ACA degradation of M2 and M3 exceeds 4%, reflecting the key role of adaptive parameter decomposition and multi-dimensional feature screening in noisy signal reconstruction. Although the sliding sampling and attention mechanism module has a relatively small reduction (ACA reduction of 3.24%, 3.77%), it also verifies its unique value in capturing complete fault information and adaptive weighting of key features. From the perspective of operational efficiency, the inference time of the model is positively correlated with performance. After removing the core feature module, the inference time is significantly reduced, but at the cost of significant performance loss. The signal processing and attention module only brings a small increase in computational complexity, achieving a reasonable balance between performance and efficiency.

**Table 3:** Comparison results of ablation experiment performance.

Model number	Average accuracy (ACA)/%	Average F1 score	Average inference time per sample (ms)	The decrease in ACA/%	The decrease in average F1 value/%
M0	94.50	0.938	18.65	--	--
M1	91.26	0.905	16.32	3.24	3.52
M2	89.75	0.889	15.87	4.75	5.22
M3	90.12	0.892	16.05	4.38	4.90
M4	87.36	0.864	12.58	7.14	7.89
M5	86.89	0.859	13.10	7.61	8.42
M6	90.83	0.898	14.26	3.77	4.26

### Robust noise suppression mechanism

To further elucidate the core mechanism of the proposed method in improving robustness under random noise, high-frequency switch harmonics, transient pulse interference, and multi noise superposition environments, the suppression of noise and enhancement of fault characteristics by each module of the method are analyzed. At the same time, a comparison of the average accuracy of fault classification under different signal-to-noise ratios (5dB, 10dB, 15dB, 20dB) is supplemented (see Table 4) to quantitatively verify the anti-noise performance of the method.

**Table 4:** Average accuracy of fault classification under different signal-to-noise ratios (%).

Mode	${\varTheta _{{\mathrm{ACA}}}}$/%			
	SNR=5dB	SNR=10dB	SNR=15dB	SNR=20dB
Island	88.25	94.20	95.63	96.12
Grid connected	86.78	93.10	94.57	95.24

According to Table 4, even in a strong noise environment of 5dB, the proposed method still achieves an average accuracy of 88.25% and 86.78% in islanding mode and grid connected mode, respectively. As the signal-to-noise ratio increases, the classification accuracy gradually improves, and in low to medium noise environments of 10dB and above, the accuracy remains stable at over 93%, fully verifying the strong robustness of the proposed method under different noise conditions. The core reason is that: (1) the sliding sampling process has noise suppression and feature preservation functions. Sliding sampling achieves complete capture of transient fault information by setting overlapping areas, reasonable window lengths, and sliding step sizes. Even if some sampling windows have strong noise interference, adjacent overlapping windows can still supplement fault feature information, avoiding feature loss caused by single window noise pollution. (2) SVMD optimization decomposition and multi-index IMF screening have precise denoising capabilities. SVMD optimizes the penalty factor through MIC to achieve adaptive bandwidth decomposition of signals. Random noise is mostly broadband low-energy noise, which is decomposed into ineffective IMFs with low CC, high SpEn, and low TKE. Only effective IMFs containing fault features are retained for signal reconstruction, achieving the goal of “denoising without losing features”. (3) The CNN-BiLSTM Attention model has feature enhancement and noise suppression functions. The convolutional and normalization layers of CNN can extract local time-frequency features of fault signals while suppressing the interference of high-frequency noise; BiLSTM captures the contextual relationship before and after fault signals through bidirectional sequence learning, which compensates for the problem of CNN’s insufficient representation of sequence features; The Attention mechanism, as the core weak feature enhancement module, will adaptively weight the key features at the time of fault occurrence, reduce the weight proportion of noise features, and make the model more focused on the core fault features, thereby improving the anti-noise ability of the classification model.

### Multi scenario application analysis

References^24,25^ and the method proposed in this paper are all focused on the application of intelligent algorithms in power equipment state monitoring and fault analysis. Through in-depth analysis of the differences between references^24,25^ and the methods proposed in this paper, it can be found that the three can actually form complementary research forms of “fault classification life prediction”, “supervised semi supervised”, and “centralized distributed”. In this regard, drawing on the methodological ideas of references^24,25^ important technical inspirations have been provided for subsequent research in this paper: (1) the semi supervised learning approach of reference^24^ solves the problem of scarce fault annotation data in practical engineering, breaks through the dependence of fully supervised learning on a large number of annotated samples, and provides a new perspective on sample utilization for the engineering implementation of fault diagnosis in DC microgrids; (2) Reference^24^ combines self-attention mechanism with variational autoencoder in feature modeling, providing a new approach for optimizing the existing attention mechanism in this paper, which can further enhance the reconstruction and representation capabilities of fault features; (3) The passive domain adaptation and federated learning method proposed in reference^25^ accurately solves the domain offset problem across operating conditions, topologies, and nodes, which is in line with the practical engineering characteristics of multi topology, multi operation mode, and distributed deployment of DC microgrids. It provides core technical support for improving the generalization of the method proposed in this paper.

On the basis of the inspiration from the above literature, the follow-up research and improvement directions of this article are as follows: (1) Drawing on the semi supervised learning framework of reference^24^, combining the variational autoencoder with the CNN-BiLSTM Attention model proposed in this article, a semi supervised fault diagnosis model is constructed, which utilizes a large amount of unlabeled normal operation data and a small amount of labeled fault data in DC microgrids to improve the diagnostic performance of the model in practical engineering scenarios with scarce samples; (2) Absorbing the technical approach of combining self-attention and feature reconstruction from reference^24^, optimizing the weighting method of the existing attention mechanism in this paper, and combining the weighting of attention features with the feature reconstruction of the variational autoencoder to further enhance the model’s ability to capture weak fault features; (3) Referring to the passive domain adaptation and federated learning method in reference^25^, a cross domain fault diagnosis model is constructed under the federated learning framework to address the domain offset problem of multi topology and multi operation modes in DC microgrids. This model achieves distributed fault diagnosis of multi node DC microgrids and improves the model’s cross domain generalization ability under different topologies and operating conditions through domain adaptive feature alignment, making the method more suitable for the distributed deployment requirements of practical engineering.

## Conclusion

This article proposes a fault detection method that combines sliding sampling, successive variational mode decomposition (SVMD), and CNN-BiLSTM-Attention model to address the issues of insufficient sensitivity and weak discriminative ability in fault signal diagnosis of DC microgrids. By effectively capturing transient fault information through sliding sampling and decomposing the fault signal using SVMD, the maximum mutual information coefficient (MIC) is used to optimize the penalty factor, and effective mode components (IMF) are selected by combining correlation coefficient, spectral entropy, and Teager Kaiser energy ratio, achieving signal denoising and reconstruction; A CNN-BiLSTM-Attention classification model was constructed, which utilizes CNN to extract local time-frequency features, BiLSTM to capture sequence context relationships, and adaptively weights key fault features through attention mechanism, effectively suppressing noise interference and improving classification accuracy. The experimental results indicate that its robustness and adaptability have been verified.

Although the method proposed in this article has achieved significant results in fault detection of DC microgrids, there are still some issues worth further exploration: (1) The topology and operating modes of actual DC microgrid systems are complex and diverse. Future research can further consider more practical fault feature extraction and classification problems under operating conditions to improve the universality of the method. (2) With the continuous growth of new energy generation scale, the types and characteristics of faults in DC microgrids may also change. How to update and optimize fault diagnosis models in real time to adapt to the new challenges brought by the improvement of new energy consumption capacity is also an important direction for future research. (3) The combination of edge computing and Internet of Things technology to realize real-time monitoring and early warning of DC microgrid faults will help further improve the operational reliability and security of the microgrid system.

## Data Availability

All data generated or analysed during this study are included in this published article.
